# Comparison of Acetaminophen (Paracetamol) With Ibuprofen for Treatment of Fever or Pain in Children Younger Than 2 Years: A Systematic Review and Meta-analysis

**DOI:** 10.1001/jamanetworkopen.2020.22398

**Published:** 2020-10-30

**Authors:** Eunicia Tan, Irene Braithwaite, Christopher J. D. McKinlay, Stuart R. Dalziel

**Affiliations:** 1 Department of Surgery, Faculty of Medical and Health Sciences, The University of Auckland, Auckland, New Zealand; 2 Emergency Department, Middlemore Hospital, Auckland, New Zealand; 3 Medical Research Institute of New Zealand, Wellington, New Zealand; 4 Liggins Institute, The University of Auckland, Auckland, New Zealand; 5 Kidz First Neonatal Care, Middlemore Hospital, Auckland, New Zealand; 6 Department of Paediatrics: Child and Youth Health, Faculty of Medical and Health Sciences, The University of Auckland, Auckland, New Zealand; 7 Children’s Emergency Department, Starship Children’s Hospital, Auckland, New Zealand

## Abstract

**Importance:**

Acetaminophen (paracetamol) and ibuprofen are the most widely prescribed and available over-the-counter medications for management of fever and pain in children. Despite the common use of these medications, treatment recommendations for young children remain divergent.

**Objective:**

To compare acetaminophen with ibuprofen for the short-term treatment of fever or pain in children younger than 2 years.

**Data Sources:**

Systematic search of the databases MEDLINE, Embase, CINAHL, and the Cochrane Central Register of Controlled Trials and the trial registers ClinicalTrials.gov and the Australian New Zealand Clinical Trials Registry from inception to March 2019, with no language limits.

**Study Selection:**

Studies of any design that included children younger than 2 years and directly compared acetaminophen with ibuprofen, reporting antipyretic, analgesic, and/or safety outcomes were considered. There were no limits on length of follow-up.

**Data Extraction and Synthesis:**

Following the Preferred Reporting Items for Systematic Reviews and Meta-analyses (PRISMA) guideline, 2 authors independently extracted data and assessed quality. Data were pooled using a fixed-effects method if *I*^2^ was less than 50% and using a random-effects method if *I*^2^ was 50% or greater.

**Main Outcomes and Measures:**

The primary outcomes were fever or pain within 4 hours of treatment onset. Safety outcomes included serious adverse events, kidney impairment, gastrointestinal bleeding, hepatotoxicity, severe soft tissue infection, empyema, and asthma and/or wheeze.

**Results:**

Overall, 19 studies (11 randomized; 8 nonrandomized) of 241 138 participants from 7 countries and various health care settings (hospital-based and community-based) were included. Compared with acetaminophen, ibuprofen resulted in reduced temperature at less than 4 hours (4 studies with 435 participants; standardized mean difference [SMD], 0.38; 95% CI, 0.08-0.67; *P* = .01; *I*^2^ = 49%; moderate quality evidence) and at 4 to 24 hours (5 studies with 879 participants; SMD, 0.24; 95% CI, 0.03-0.45; *P* = .03; *I*^2^ = 57%; moderate-quality evidence) and less pain at 4 to 24 hours (2 studies with 535 participants; SMD, 0.20; 95% CI, 0.03-0.37; *P* = .02; *I*^2^ = 25%; moderate-quality evidence). Adverse events were uncommon. Acetaminophen and ibuprofen appeared to have similar serious adverse event profiles (7 studies with 27 932 participants; ibuprofen vs aceteminophen: odds ratio, 1.08; 95% CI, 0.87-1.33; *P* = .50, *I*^2^ = 0%; moderate-quality evidence).

**Conclusions and Relevance:**

In this study, use of ibuprofen vs acetaminophen for the treatment of fever or pain in children younger than 2 years was associated with reduced temperature and less pain within the first 24 hours of treatment, with equivalent safety.

## Key Points

**Question:**

Are there differences in the antipyretic, analgesic, and safety profiles of acetaminophen (paracetamol) compared with ibuprofen for the short-term treatment of fever or pain in children younger than 2 years?

**Findings:**

In this meta-analysis of 19 studies with 241 138 participants, ibuprofen, compared with acetaminophen, was associated with reduced temperature at less than 4 hours and 4 to 24 hours and less pain at 4 to 24 hours. Adverse events were uncommon.

**Meaning:**

In this study, use of ibuprofen vs acetaminophen for the treatment of fever or pain in children younger than 2 years was associated with reduced temperature and less pain within the first 24 hours of treatment, with equivalent safety.

## Teaser

This systematic review and meta-analysis compares acetaminophen with ibuprofen for the short-term treatment of fever or pain in children younger than 2 years.

## Introduction

Acetaminophen (paracetamol) and ibuprofen are the most widely prescribed and available over-the-counter medications for management of fever and pain in children. Data from a prospective cohort of 6476 children followed from birth^1^ suggest that as many as 95% of children are exposed to acetaminophen by age 9 months. Despite the common use of these medications, treatment recommendations for young children remain divergent, especially among those younger than 6 months.^2^ While acetaminophen is uniformly recommended across countries for use from the neonatal period, the maximum daily dose beyond the neonatal period varies from 60 mg/kg/d in New Zealand^3^ and the United Kingdom^4^ to 90 mg/kg/d in the United States.^5^ Recommendations for ibuprofen use for the treatment of fever and pain in young children are considerably more variable. The New Zealand Formulary for Children,^3^ based on the British National Formulary for Children,^4^ recommends ibuprofen use from age 1 month at 5 mg/kg/dose, 3 to 4 times daily, to a maximum dose of 30 mg/kg/d. In contrast, in the United States, ibuprofen use is only recommended for children aged at least 6 months, with a higher maximum daily dose of 40 mg/kg/d.^5^

Several authors caution against the use of ibuprofen in younger infants, citing a higher risk of acute kidney injury, particularly in the context of dehydration.^2,6,7,8^ Epidemiological studies conducted in North America, the United Kingdom, and Europe suggest that ibuprofen may play a role in the development of serious bacterial infection. Case-control and prospective cohort studies have shown a 2-fold to 5-fold increase in the odds of developing soft tissue infection with ibuprofen use in the context of primary varicella infection,^9,10,11,12^ and a similar increase in the odds of developing empyema with ibuprofen use during treatment of community-acquired pneumonia.^13,14,15,16^ Although acetaminophen is often considered first-line in the treatment of fever and pain in children because its safety is perceived to be more assured,^6,17^ there is growing evidence suggesting acetaminophen use in children is associated with an increased risk of developing asthma and related atopic disease.^18,19,20,21^ Data regarding the risks of acetaminophen or ibuprofen in young children are often incorporated in studies across the pediatric age spectrum, but specific literature concerning the risk of serious adverse events (SAEs) and systematic reviews of efficacy and safety in children younger than 2 years, particularly in infants younger than 6 months, are lacking.

Previous systematic reviews comparing the efficacy and safety of acetaminophen with ibuprofen in children have shown ibuprofen to be at least as efficacious as acetaminophen as an analgesic and more efficacious as an antipyretic,^22,23,24^ with no differences in safety.^22,23,24,25^ However, there is considerable heterogeneity among the individual studies in terms of population, with children ranging from age 1 month to 18 years, limiting the applicability of the evidence to younger children. To date, only 1 nonsystematic review has addressed the efficacy and safety of ibuprofen in younger children.^2^ Ziesenitz et al^2^ concluded that short-term use of ibuprofen for the treatment of fever or pain is safe in infants older than 3 to 6 months with a body weight greater than 5 to 6 kg when special attention is given to their hydration status.^2^ However, this review did not compare the relative efficacy and safety of acetaminophen with ibuprofen in infants and young children.

The aim of this systematic review was to compare the antipyretic, analgesic, and safety profiles of acetaminophen with ibuprofen for the short-term treatment of fever or pain in children younger than 2 years. A secondary aim was to compare the safety of acetaminophen with ibuprofen for short-term treatment of fever or pain in infants younger than 6 months.

## Methods

This systematic review was conducted in accordance with the Preferred Reporting Items for Systematic Reviews and Meta-analyses (PRISMA) reporting guideline. The protocol was registered in PROSPERO (CRD42019121311).

### Search Strategy

We searched MEDLINE, Embase, CINAHL, and the Cochrane Central Register of Controlled Trials (CENTRAL) databases from inception to March 2019 using key words, medical subject heading terms, and Emtree headings, including *paracetamol*, *acetaminophen*, *ibuprofen*, *newborn*, *neonate*, *infant*, *baby*, *child*, *toddler*, and *pediatric*, with spelling variants. The search was limited to human studies, with no limits on language or year of publication. We searched trial registers ClinicalTrials.gov and the Australian New Zealand Clinical Trials Registry for ongoing or recently completed trials, and we hand searched reference lists of included studies and review papers. One author (E.T.) conducted the search and initial title/abstract screening. Two authors (E.T. and I.B.) independently assessed full-text reports for eligibility. Screening and eligibility assessments were performed using Covidence.

### Inclusion Criteria

We included all published studies (trials, cohort, case-control) from any health care setting or country that compared short-term use of acetaminophen with ibuprofen for fever or pain in children younger than 2 years and reported 1 or more primary or secondary outcomes. Studies whose population included participants older than 2 years were included if they published data for the age group younger than 2 years or if study authors provided unpublished data via personal communication. If study authors did not provide additional data, we included studies if more than 50% of the study population was younger than 2 years. We included studies with both short-term and long-term follow-up. We excluded case series and studies in which there was medication cointervention.

### Data Extraction and Analysis

Two authors (E.T. and I.B.) independently extracted data using a prespecified form. The primary outcomes were fever (continuous variable) or pain within 4 hours of treatment onset. Secondary outcomes included fever (categorical variable) within 4 hours and fever or pain at 4 to 24 hours, 1 to 3 days, and more than 3 days. Secondary safety outcomes were measured at 28 days or less and more than 28 days and included SAEs; kidney impairment; gastrointestinal (GI) bleeding; hepatoxicity; severe soft tissue infection; empyema; and asthma and/or wheeze, as defined by study authors. If studies reported more than 1 data point within a period, we extracted the data that occurred at the earliest point within that period, except for secondary safety outcomes at more than 28 days, in which case we extracted the longest-term data that were reported.

### Quality of Evidence

Two authors (E.T. and I.B.) independently assessed the risk of bias for each study using the Cochrane Risk-of-Bias 2 tool for randomized trials,^26^ the Risk of Bias in Nonrandomized Studies–of Interventions tool for nonrandomized studies of interventions,^27^ and the overall quality of evidence for each outcome using the Grading of Recommendations Assessment, Development and Evaluation (GRADE) approach.^28^ Disagreements during the review process were resolved through discussion or by consultation with a third author (C.J.D.M. or S.R.D.).

### Statistical Analysis

Data from randomized and nonrandomized studies were analyzed separately on an intention-to-treat basis. Meta-analysis was performed separately for continuous and categorical outcomes for fever and pain and for secondary safety outcomes, using Review Manager version 5.3.5 (RevMan). Heterogeneity between studies was calculated using the *I*^2^ statistic in RevMan. An inverse variance, fixed-effects method was used if *I*^2^ was less than 50%, and a random-effects method was used if *I*^2^ was 50% or greater. Exposure effects are presented as standardized mean difference (SMD) or odds ratio (OR), using adjusted results when available, with 95% CIs. A 2-tailed *P* < .05 was considered statistically significant. If meta-analysis was not possible, a narrative synthesis is provided.

We planned subgroup analyses for primary outcomes by dose (ie, lower vs higher dosages of acetaminophen [≤10 mg/kg vs >10 mg/kg] and ibuprofen [≤5 mg/kg vs >5 mg/kg]), age (<6 months vs ≥6 months), indication (primary varicella infection vs not primary varicella infection), and outcome assessment (analgesic effect assessed by parent or caregiver vs health professional). We planned a sensitivity analysis, excluding studies at high risk of bias.

## Results

### Search Results

**Figure 1.**
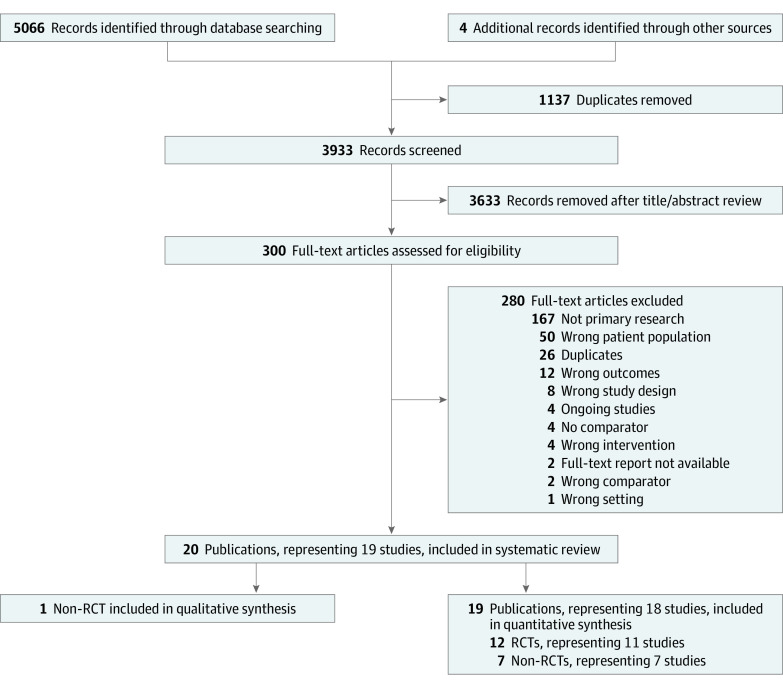
Flowchart of Study Identification, Inclusion, and Exclusion RCT indicates randomized clinical trial.

Of 3933 records identified, 3633 were excluded following title and abstract screening, 276 were excluded following full-text screening, and 4 were ongoing studies. Thus, 19 studies (20 publications^12,29,30,31,32,33,34,35,36,37,38,39,40,41,42,43,44,45,46,47^) were included (Figure 1).

### Characteristics of the Included Studies

Overall, 11 studies (12 publications^29,30,31,32,33,34,35,36,37,38,39,40^) reported data from randomized studies involving 28 450 participants (eTable 1 in the Supplement). Of these, 9 reported fever outcomes,^29,30,32,33,34,35,36,39,40^ 4 reported pain outcomes,^33,35,39,40^ and 9 (10 publications^30,31,32,33,35,36,37,38,39,40^) reported safety outcomes.

Overall, 8 studies^12,41,42,43,44,45,46,47^ reported data from nonrandomized studies involving 212 688 participants (eTable 1 in the Supplement). Of these, 2 reported fever outcomes,^42,43^ 0 reported pain outcomes, and 8 reported safety outcomes.^12,41,42,43,44,45,46,47^ Data from 1 study^45^ could not be included in the quantitative synthesis. Overall, 6 of 8 nonrandomized studies^12,41,44,45,46,47^ had author adjustment for potential confounding, but only the outcomes of asthma and/or wheeze were extractable; all other extracted effect estimates were unadjusted.

The studies were conducted in the United States, the United Kingdom, France, the Netherlands, Israel, Turkey, and Iran. They took place in various clinical settings, including pediatric and mixed emergency departments, pediatric wards, hospital-based clinics, and community clinics (eTable 1 in the Supplement).

Participants ranged in age from birth to 18 years. A total of 4 studies^33,45,46,47^ with 198 049 participants had a population that was exclusively younger than 2 years, 3 studies^38,42,43^ with 27 188 participants published data for participants younger than 2 years, 4 studies^12,31,34,36^ with 263 participants provided unpublished data for participants younger than 2 years, and, in 8 studies^29,30,32,35,39,40,41,44^ with 15 638 participants, more than 50% of the study population was younger than 2 years. Four studies^38,39,41,45^ with 43 498 participants included infants younger than 6 months; 2 studies^38,45^ with 29 067 participants included 2465 (8.5%) younger than 6 months, and 2 studies^39,41^ with 14 431 participants had an unknown proportion of participants younger than 6 months.

Two randomized studies were at high risk of bias. All nonrandomized studies were at moderate or serious risk of bias (eTable 2 in the Supplement).

### Primary Outcomes

**Figure 2.**
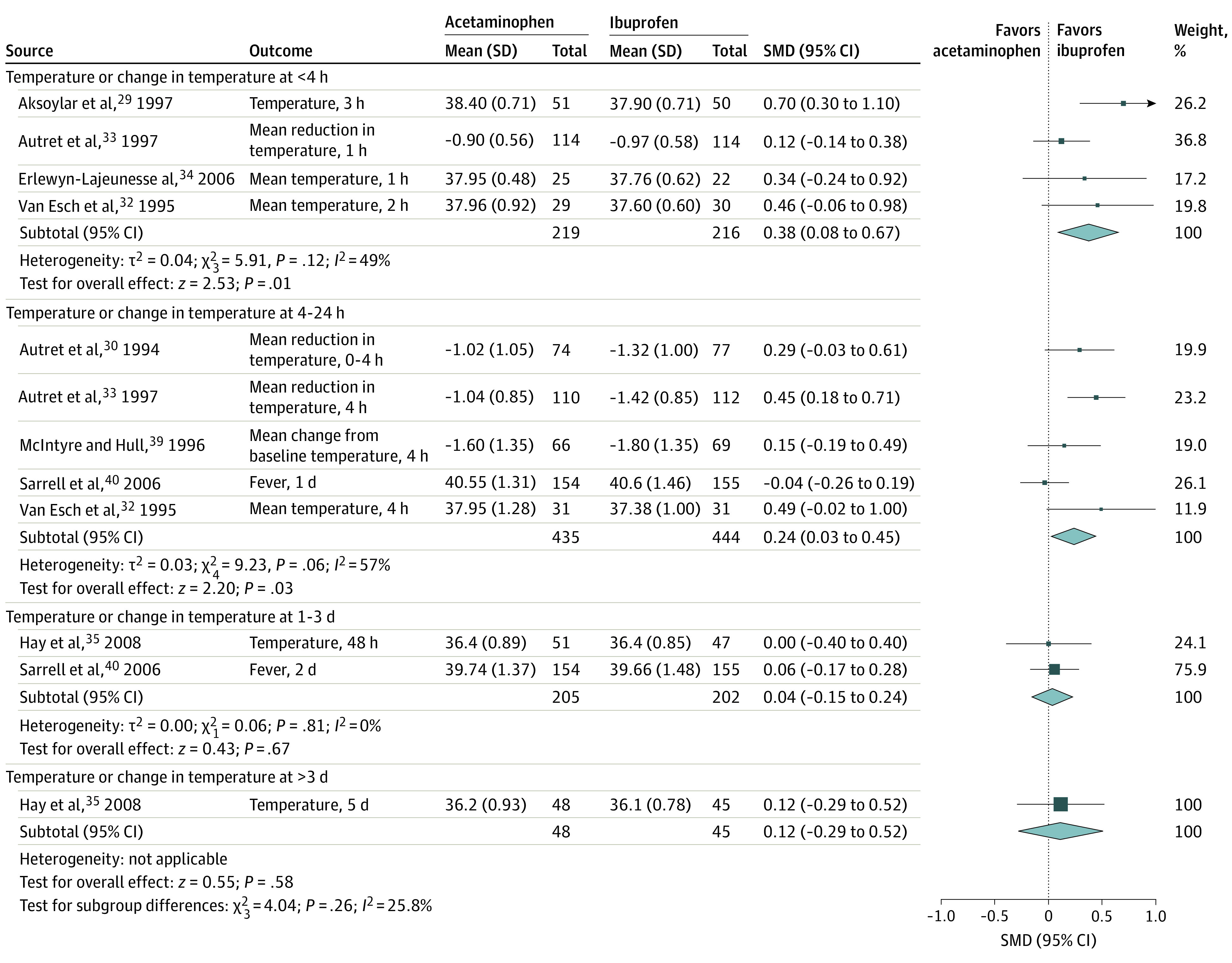
Antipyretic Profile of Ibuprofen vs Acetaminophen From Randomized Studies with Continuous Variables SMD indicates standardized mean difference.

Moderate-quality evidence from randomized studies showed that compared with acetaminophen, ibuprofen was associated with reduced temperature within 4 hours (4 studies^29,32,33,34^ with 435 participants; SMD, 0.38; 95% CI, 0.08 to 0.67; *P* = .01; *I*^2^ = 49%) (Table and Figure 2). Very low-quality evidence from nonrandomized studies showed that ibuprofen and acetaminophen had similar antipyretic profiles within 4 hours (2 studies^42,43^ with 123 participants; SMD, −0.04; 95% CI, −0.40 to 0.31; *P* = .81; *I*^2^ = 0%) (Table; eFigure 1 in the Supplement). None of the included studies reported pain outcomes within 4 hours from treatment onset. Subgroup analyses for fever reduction within 4 hours comparing lower and higher dosages of ibuprofen and acetaminophen did not alter the results (eFigure 2 in the Supplement). Data were not available for the remainder of the planned subgroup analyses. In sensitivity analysis, exclusion of 2 studies at high risk of bias^29,43^ did not alter the results.

**Table.** Grading of Recommendations Assessment, Development and Evaluation Summary of Quality of Evidence for Antipyretic, Analgesic, and Safety Outcomes

Outcome	Time	Study type	Participants (studies), No.	Exposure effect, ibuprofen vs acetaminophen	Quality of evidence
Primary outcomes					
Reduced temperature	<4 h	RCT	435 (4)	SMD, 0.38 (95% CI, 0.08 to 0.67)	Moderate^a^
	<4 h	Non-RCT	123 (2)	SMD, −0.04 (95% CI,−0.40 to 0.31)	Very low^a^^,^^b^
Reduced pain	<4 h	RCT	0 (0)	NA	No evidence
	<4 h	Non-RCT	0 (0)	NA	No evidence
Secondary outcomes					
Afebrile	<4 h	RCT	587 (5)	OR, 1.86 (95% CI, 1.01 to 3.44)	Moderate^a^^,^^c^
Serious adverse events	≤28 d	RCT	27 932 (7)	OR, 1.08 (95% CI, 0.87 to 1.33)	Moderate^a^
	≤28 d	Non-RCT	14 364 (2)	Not estimable	Very low^a^^,^^b^
Kidney impairment	≤28 d	RCT	27 753 (4)	OR, 0.97 (95% CI, 0.44 to 2.15)	Moderate^a^
	≤28 d	Non-RCT	14 281 (1)	Not estimable	Very low^a^^,^^b^
Gastrointestinal bleeding	≤28 d	RCT	27 531 (3)	OR, 3.56 (95% CI, 0.18 to 68.97)	Low^a^^,^^b^
	≤28 d	Non-RCT	14 281 (1)	Not estimable	Very low^a^^,^^b^
Hepatotoxicity	≤28 d	RCT	466 (2)	OR, 0.49 (95% CI, 0.09 to 2.72)	Moderate^a^
	≤28 d	Non-RCT	0 (0)	NA	No evidence
Soft tissue infection	≤28 d	RCT	157 (1)	Not estimable	Low^a^^,^^b^
	≤28 d	Non-RCT	14 290 (2)	OR, 12.60 (95% CI, 0.45 to 356.39)	Very low^a^^,^^b^^,^^c^
Empyema	≤28 d	RCT	157 (1)	Not estimable	Low^a^^,^^b^
	≤28 d	Non-RCT	0 (0)	NA	No evidence
Asthma and/or wheeze	≤28 d	RCT	27 372 (3)	OR, 0.83 (95% CI, 0.51 to 1.37)	Moderate^a^
	≤28 d	Non-RCT	57 974 (2)	OR, 0.98 (95% CI, 0.74 to 1.30)	Very low^a^^,^^b^

Abbreviations: NA, not applicable; OR, odds ratio; RCT, randomized clinical trial; SMD, standardized mean difference.

^a^ Downgraded for risk of bias.

^b^ Downgraded for imprecision.

^c^ Downgraded for heterogeneity.

### Secondary Outcomes

#### Fever

##### Data From Randomized Studies

For continuous fever outcomes (Figure 2), moderate-quality evidence showed that, compared with acetaminophen, ibuprofen was associated with reduced temperature at 4 to 24 hours from treatment onset (5 studies^30,32,33,39,40^ with 879 participants; SMD, 0.24; 95% CI, 0.03 to 0.45). Moderate to low-quality evidence showed that ibuprofen and acetaminophen had similar antipyretic profiles at 1 to 3 days (2 studies^35,40^ with 407 participants; SMD, 0.04; 95% CI, −0.15 to 0.24) and at more than 3 days (1 study^35^ with 93 participants; SMD, 0.12; 95% CI, −0.29 to 0.52).

**Figure 3.**
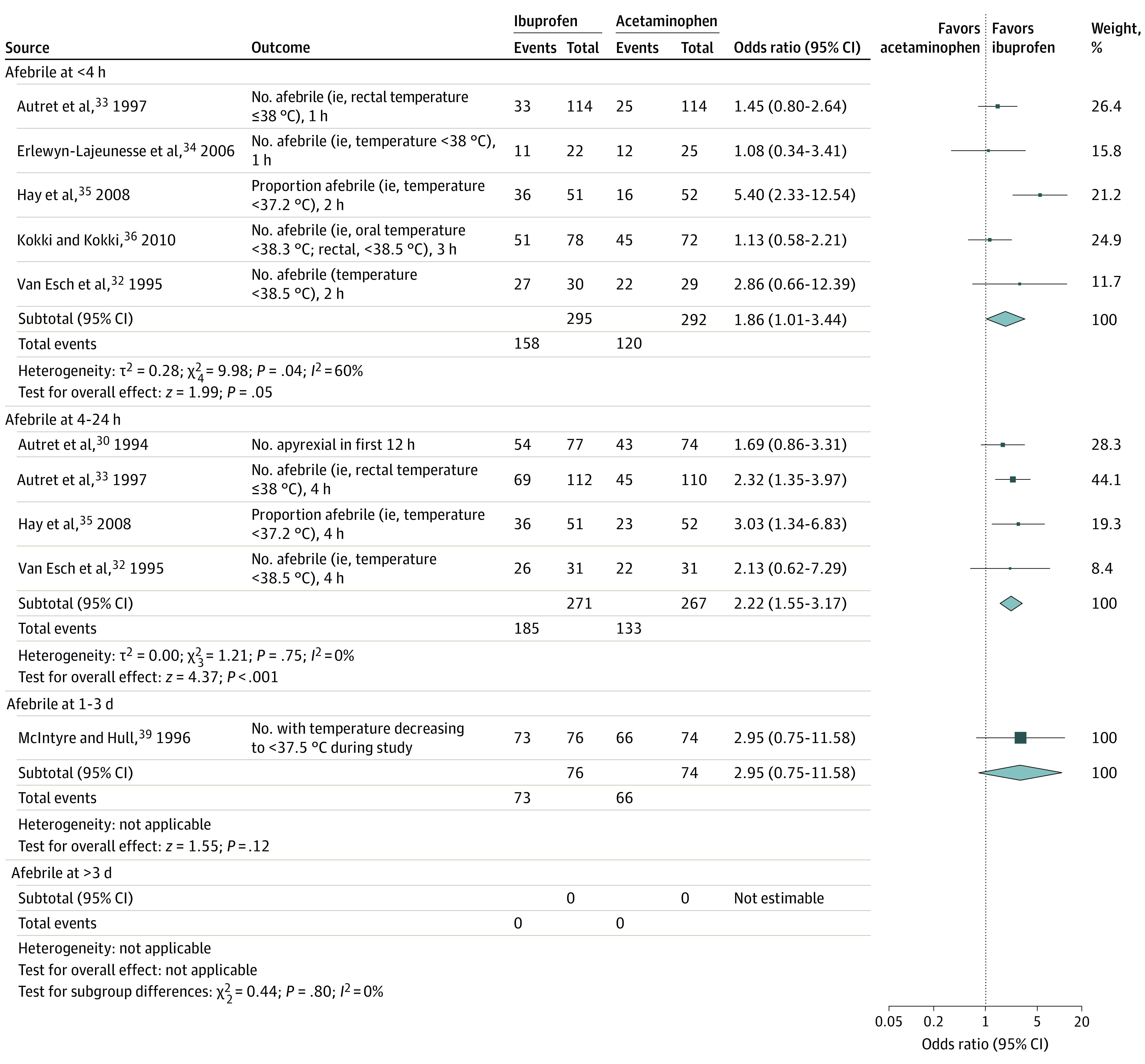
Antipyretic Profile of Ibuprofen vs Acetaminophen From Randomized Studies with Categorical Variables

For categorical fever outcomes (Figure 3), moderate-quality evidence showed that children treated with ibuprofen were more likely to be afebrile within 4 hours (5 studies^32,33,34,35,36^ with 587 participants; ibuprofen, 158 of 295 [53.6%] vs acetaminophen, 120 of 292 [41.1%]) and at 4 to 24 hours from treatment onset (4 studies^30,32,33,35^ with 538 participants; ibuprofen, 185 of 271 [68.3%] vs acetaminophen, 133 of 267 [49.8]) (Table), but with no difference at 1 to 3 days (1 study^39^ with 150 participants; ibuprofen, 73 of 76 [96.1%] vs acetaminophen, 66 of 74 [89.2%]). Data were not available after more than 3 days.

##### Data From Nonrandomized Studies

For continuous fever outcomes (eFigure 1 in the Supplement), very low-quality evidence from 1 study^42^ with 40 participants showed that ibuprofen and acetaminophen had similar antipyretic profiles at 4 to 24 hours (SMD, 0.18; 95% CI, −0.45 to 0.80).^42^ Data were not available after more than 24 hours. None of the nonrandomized studies reported categorical fever outcomes.

#### Pain

**Figure 4.**
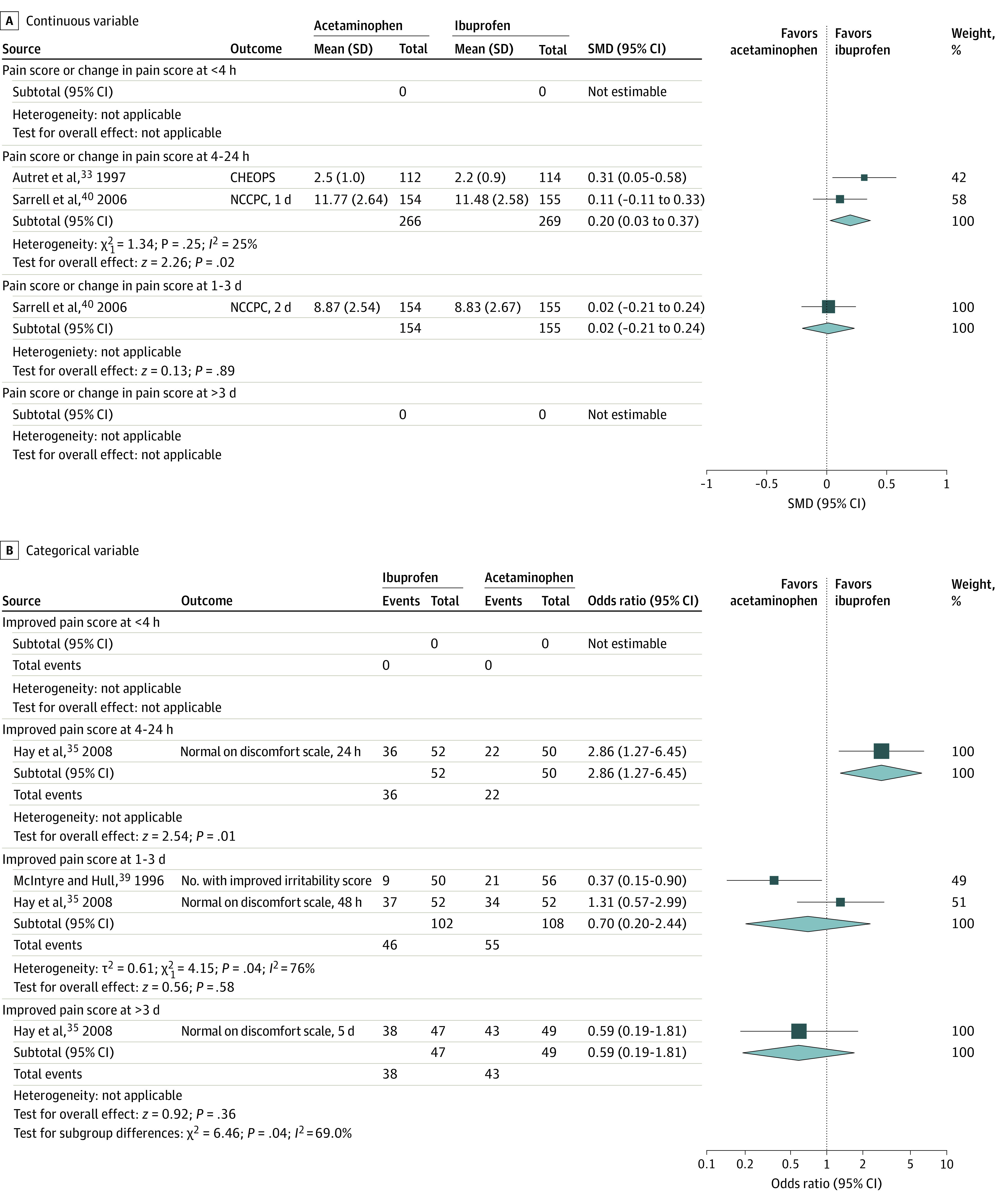
Analgesic Profile of Ibuprofen vs Acetaminophen From Randomized Studies SMD indicates standardized mean difference.

For continuous pain outcomes (Figure 4A), moderate-quality evidence showed that compared with acetaminophen, ibuprofen was associated with less pain at 4 to 24 hours from treatment onset (2 studies^33,40^ with 535 participants; SMD, 0.20; 95% CI, 0.03 to 0.37; *P* = .02). Low-quality evidence from 1 study with 299 participants showed that ibuprofen and acetaminophen had similar analgesic profiles at 1 to 3 days (SMD, 0.02; 95% CI, −0.21 to 0.24).^40^ Data were not available after more than 3 days.

For categorical pain outcomes (Figure 4B), low-quality evidence from 1 study^35^ with 102 participants showed that children treated with ibuprofen were more likely to be pain free at 4 to 24 hours from treatment onset (ibuprofen, 36 of 52 [69.2%] vs acetaminophen, 22 of 50 [44.0%]).^35^ Low-quality evidence showed that children treated with ibuprofen and acetaminophen had similar analgesic profiles at 1 to 3 days (2 studies^35,39^ with 210 participants; ibuprofen, 46 of 102 [45.1%] vs acetaminophen, 55 of 108 [50.9%]) and at more than 3 days (1 study^35^ with 96 participants; ibuprofen, 38 of 47 [80.9%] vs acetaminophen, 43 of 49 [87.8%]). None of the nonrandomized studies reported pain outcomes.

#### Safety

##### Data From Randomized Studies

There were very low rates of adverse events reported across all studies, with most studies reporting 0 adverse events during their follow-up period. For short-term (ie, ≤28 days) safety outcomes (Table; eFigure 3 in the Supplement), moderate-quality evidence from 7 studies^30,32,33,35,36,38,39^ with 27 932 participants showed that children taking ibuprofen or acetaminophen had similar likelihood of SAEs (ibuprofen, 264 of 18 371 [1.4%] vs acetaminophen, 126 of 9561 [1.3%]; OR, 1.08; 95% CI, 0.87-1.33; *P* = .50; *I*^2^ = 0%). Of these, 4 studies^30,32,33,39^ with 606 participants had no SAEs in either treatment group. Moderate-quality evidence showed that children taking ibuprofen or acetaminophen had similar likelihood of kidney impairment (3 studies^36,37,38,40^ with 27 753 participants; 19 of 18 326 [0.1%] vs 11 of 9427 [0.1%]), hepatotoxicity (2 studies^36,40^ with 466 participants; 2 of 233 [0.9%] vs 4 of 233 [1.7%]) and asthma and/or wheeze (3 studies^36,38,39^ with 27 372 participants; 41 of 18 092 [0.2%] vs 26 of 9280 [0.3%]) (Table; eFigure 3 in the Supplement).

Low-quality evidence showed that children taking ibuprofen or acetaminophen had similar likelihood of GI bleeding (3 studies^36,38,40^ with 27 531 participants; 3 of 18 171 [0.02%] vs 0 of 9360) (Table; eFigure 3 in the Supplement). Only 1 study^36^ with 157 participants reported outcomes for severe soft tissue infection and empyema, recording no events in either treatment arm.

Only 2 studies^31,40^ with 354 participants reported long-term (ie, >28 days) safety outcomes (eFigure 3 in the Supplement).^31,40^ Data from 1 study^31^ with 45 participants showed that children taking ibuprofen or acetaminophen had similar likelihood of asthma and/or wheeze (15 of 26 [57.7%] vs 12 of 19 [63.2%]).^31^ There were no SAEs (0 of 354 participants), kidney impairment (0 of 354 participants), GI bleeding (0 of 354 participants), hepatoxicity (0 of 354 participants), severe soft tissue infection (0 of 45 participants), or empyema (0 of 45 participants) reported.

##### Data From Nonrandomized Studies

Five studies^12,41,43,44,46^ with 72 347 participants reported short-term (ie, ≤28 days) safety outcomes (Table; eFigure 4 in the Supplement). There were no events reported for SAEs (0 of 14 364 participants), kidney impairment (0 of 14 281 participants), or GI bleeding (0 of 14 281 participants).^41,43^ Very low-quality evidence showed that children taking ibuprofen or acetaminophen had similar likelihood of developing severe soft tissue infection (2 studies^12,41^ with 14 290 participants; 4 of 7387 [0.1%] vs 0 of 6903) and asthma and/or wheeze (2 studies^44,46^ with 57 974 participants; 22 of 8997 [0.2%] vs 39 of 48 977 [0.1%]).

Only 3 nonrandomized studies^42,46,47^ with 195 855 participants reported long-term (>28 days) safety outcomes (eFigure 4 in the Supplement). There were no SAEs (0 of 40 participants),^42^ and very low-quality evidence showed that children taking ibuprofen or acetaminophen had similar likelihood of kidney impairment (1 study^47^ with 138 299 participants; 0 of 1724 vs 61 of 136 575 [0.04%]), GI bleeding (1 study^47^ with 138 299 participants; 6 of 1,724 [0.3%] vs 471 of 136 575 [0.3%]) and developing severe soft tissue infection (1 study^47^ with 138 299 participants; 0 of 1724 vs 5 of 136 575 [0.004%]). Very low-quality evidence showed that children taking ibuprofen had lower odds of developing asthma and/or wheeze compared with children taking acetaminophen (1 study^46^ with 37 729 participants; adjusted OR, 0.83; 95% CI, 0.72-0.97; *P* = .02). Data were not available from nonrandomized studies for short-term or long-term outcomes of hepatotoxicity and empyema.

### Studies Not Included in Quantitative Synthesis

Sordillo et al^45^ investigated the associations between intake of either acetaminophen or ibuprofen during the first year of life and asthma-related outcomes using data from 1490 mother-child pairs in Project Viva,^48^ a longitudinal prebirth cohort study with a moderate risk of bias. Adjusting for all covariates, they found no increase in likelihood of current asthma in midchildhood for higher infant acetaminophen or ibuprofen intake (eTable 1 in the Supplement).

## Discussion

Our review of acetaminophen or ibuprofen for fever or pain in children younger than 2 years found moderate-quality evidence that compared with acetaminophen, ibuprofen was associated with reduced temperature at less than 4 hours and at 4 to 24 hours and less pain at 4 to 24 hours. The superiority of ibuprofen as an antipyretic did not continue beyond 24 hours after treatment onset. No data were available on analgesic outcomes at less than 4 hours. Our review found low-quality to moderate-quality evidence that acetaminophen and ibuprofen have a similar safety profile with respect to SAEs, kidney impairment, GI bleeding, hepatotoxicity, and asthma and/or wheeze at 28 days or less. Overall, adverse events were uncommon or rare, with most studies reporting no events. These findings are supported by similar results from previous systematic reviews involving older children,^22,23,24,25^ while adding to the body of evidence on the antipyretic, analgesic, and safety profiles of acetaminophen and ibuprofen in children younger than 2 years of age.

We demonstrated a statistical benefit at less than 4 hours and at 4 to 24 hours of ibuprofen compared with acetaminophen when used for fever. Although the SMDs were small, this benefit extended to categorical outcomes with children receiving ibuprofen being approximately twice as likely to be afebrile at these points. Of note, these benefits were identified in the randomized studies, giving greater certainty to the results. However, the clinical importance of these findings is uncertain. When antipyretics are used in febrile children, the therapeutic aim is to improve the child’s overall comfort.^49^ Yet, discomfort was not universally recorded as an inclusion criterion, with only 1 randomized study^38^ having additional possible discomfort criteria. It is therefore disappointing that, despite 241 138 participants enrolled in the randomized and nonrandomized studies, data were not available for pain outcomes within 4 hours of treatment. Future studies should focus on these data. Evidence from randomized studies showed a benefit of ibuprofen in continuous and categorical pain outcomes at 4 to 24 hours, suggesting a clinical benefit at this time but not beyond. Collectively, these findings provide weak evidence to support ibuprofen use over acetaminophen.

Several authors have cautioned against the use of ibuprofen in healthy infants aged younger than 3 to 6 months due to safety concerns.^2,6,7,8^ A secondary aim of our review was to compare the safety of acetaminophen with ibuprofen for short-term treatment of fever or pain in infants younger than 6 months. Only 2 randomized studies^38,39^ in our review included infants younger than 6 months; unfortunately, there were no extractable data for this prespecified subgroup analysis. The only large-scale randomized clinical trial (RCT) that included infants younger than 6 months is the Boston Fever Study,^50^ a practitioner-based, double-masked RCT designed to assess the safety of ibuprofen suspension when used to treat fever in children. In a post hoc analysis,^38^ none of the 319 infants aged 1 to 6 months were hospitalized for acute GI bleeding, acute kidney failure, asthma, or bronchiolitis, and risk of hospitalization did not vary significantly by antipyretic assignment. Our review did not identify any studies comparing acetaminophen vs ibuprofen for fever and pain in neonates. Thus, we must be cautious of extrapolation of evidence to this age group. However, both ibuprofen and acetaminophen have been used for closure of patent ductus arteriosus in preterm infants, with little difference in safety profiles from a short course.^51,52^ Further studies that include infants younger than 6 months are needed.

A commonly cited reason for avoidance of ibuprofen in younger children is their perceived higher risk of kidney toxic effects, particularly in the context of dehydration.^6,7,8,53^ We did not find any evidence to support this view. Although we did not specifically examine the use of acetaminophen or ibuprofen in the context of illness with a risk of dehydration, 2 randomized studies^37,40^ in our analysis with 27 374 participants included children with concomitant dehydration, and neither found evidence to suggest a higher likelihood of kidney impairment in children using ibuprofen compared with acetaminophen.

Concern has been raised that ibuprofen use may increase the risk of serious bacterial infection in children, specifically, invasive group A streptococcal (GAS) skin infection in the context of primary varicella infection^6,53^ and empyema.^13^ We found insufficient evidence to support or refute these hypotheses. Only 2 randomized studies^31,36^ with 202 participants contributed data for the analysis of these outcomes; both had small sample sizes and recorded no events. Unadjusted and imprecise effect estimates of the likelihood of severe soft tissue infection were available from 3 nonrandomized studies^12,41,47^ at serious risk of bias. These results may be confounded by indication bias because ibuprofen is generally reserved for more severe illness. The only systematic review to specifically examine the risk of GAS infections with acetaminophen or ibuprofen treatment was inconclusive.^25^ Higher quality evidence from randomized trials or large well-designed prospective cohort studies is needed to address these concerns.

In agreement with previous authors,^21,25^ our systematic review found that children treated with acetaminophen or ibuprofen had no difference in the likelihood of immediately exacerbating asthma and/or wheeze. Others have found that ibuprofen use may have a protective effect in terms of asthma morbidity compared with acetaminophen up to 28 days after use,^21^ and acetaminophen use in the first year of life was found to be a risk factor for wheezing and asthma at age 6 to 7 years in an epidemiological study of 205 487 children.^19^ We found very low-quality evidence that children treated with ibuprofen may have a lower likelihood of developing asthma and/or wheeze at more than 28 days, based on 1 nonrandomized study.^46^ A randomized trial is needed to provide more conclusive evidence on the effect of acetaminophen or ibuprofen exposure on asthma morbidity in childhood. One such large study is currently under way (ACTRN 12618000303246).

### Strengths and Limitations

A strength of this review is the inclusion of several important clinical outcomes that have direct relevance to pediatric patient care. We identified both randomized and observational studies to address our review questions. Consequently, a degree of heterogeneity was found across studies with respect to study setting, sample size, drug dosages, and treatment duration. However, this diversity reflects the use of acetaminophen and ibuprofen in routine clinical practice and may strengthen the applicability of our review findings to patients with differing illnesses in various clinical settings.

A key limitation already alluded to is the small number of participants (ie, 796) who could be included in the analgesic analysis, with only 4 studies^33,35,39,40^ reporting pain outcomes, none of which reported our primary outcome of pain within 4 hours of treatment onset. Furthermore, the small sample size in many of the studies included made the comparison of adverse events difficult because of the low rates reported across most studies. Using study authors’ definitions of adverse events could have led to inconsistent adverse events data across studies. Additionally, only 9 studies^12,31,37,38,41,44,45,46,47^ investigated safety as a primary outcome, and it is possible that there is measurement bias during adverse event data collection in the remainder of the studies. Randomized studies in our review were typically short, providing limited data on adverse events at more than 28 days. Many of the long-term adverse events captured in the review were from observational studies, with their inherent biases. Thus, results of this review pertaining to safety outcomes should be interpreted accordingly.

## Conclusions

In this study, ibuprofen use was associated with reduced temperature and less pain within the first 24 hours than acetaminophen use. The lack of analgesic outcome data within 4 hours of use weakens the clinical importance of these findings. The 2 medications appear to have similar safety profiles in the short term, with very low rates of adverse events overall. The evidence regarding the risk of serious bacterial infection remains inconclusive, and there are limited data on younger infants and on longer-term adverse events. Large, randomized trials are needed to address these knowledge gaps, designed to include and report on the subgroup of infants younger than 6 months and to investigate the safety of acetaminophen and ibuprofen as a primary end point, with long-term follow-up and monitoring for adverse events.

## Notes

Supplement. **eTable 1. **Characteristics of Included Studies **eTable 2.** Risk of Bias Assessment of Included Studies **eFigure 1. **Antipyretic Profile (Continuous Variable) of Ibuprofen vs Acetaminophen From Nonrandomized Studies **eFigure 2. **Subgroup Analyses of Antipyretic Profile Within 4 Hours Comparing Lower and Higher Dosages of Ibuprofen vs Acetaminophen **eFigure 3. **Adverse Events With Ibuprofen vs Acetaminophen From Randomized Studies **eFigure 4. **Adverse Events With Ibuprofen vs Acetaminophen From Nonrandomized Studies
