# Supplementary material for: Comparison of Acetaminophen (Paracetamol) With Ibuprofen for Treatment of Fever or Pain in Children Younger Than 2 Years: A Systematic Review and Meta-analysis
Source: JAMA Netw Open. 2020 Oct 30;3(10):e2022398. doi: 10.1001/jamanetworkopen.2020.22398 (PMC7599455; doi:10.1001/jamanetworkopen.2020.22398)
Supplement: Supplement. — eTable 1. Characteristics of Included Studies eTable 2. Risk of Bias Assessment of Included Studies eFigure 1. Antipyretic Profile (Continuous Variable) of Ibuprofen vs Acetaminophen From Nonrandomized Studies eFigure 2. Subgroup Analyses of Antipyretic Profile Within 4 Hours Comparing Lower and Higher Dosages of Ibuprofen vs Acetaminophen eFigure 3. Adverse Events With Ibuprofen vs Acetaminophen From Randomized Studies eFigure 4. Adverse Events With Ibuprofen vs Acetaminophen From Nonrandomized Studies [file jamanetwopen-e2022398-s001.pdf]

Supplemental Online Content

Tan E, Braithwaite I, McKinlay CJD, Dalziel SR. Comparison of acetaminophen (paracetamol) with ibuprofen for treatment of fever or pain in children younger than 2 years: a systematic review and meta-analysis. *JAMA Netw Open*. 2020;3(10):e2022398. doi:10.1001/jamanetworkopen.2020.22398

eTable 1. Characteristics of Included Studies
eTable 2. Risk of Bias Assessment of Included Studies
eFigure 1. Antipyretic Profile (Continuous Variable) of Ibuprofen vs Acetaminophen From Nonrandomized Studies
eFigure 2. Subgroup Analyses of Antipyretic Profile Within 4 Hours Comparing Lower and Higher Dosages of Ibuprofen vs Acetaminophen
eFigure 3. Adverse Events With Ibuprofen vs Acetaminophen From Randomized Studies
eFigure 4. Adverse Events With Ibuprofen vs Acetaminophen From Nonrandomized Studies

This supplemental material has been provided by the authors to give readers additional information about their work.

© 2020 Tan E et al. *JAMA Network Open.*

eTable 1. Characteristics of Included Studies

Author and date	Study design	Setting, Country	Study population	Inclusion criteria	Exclusion criteria	Ibuprofen		Acetaminophen		Outcomes assessed	Follow-up duration	Included in meta-analysis	Adjustment for potential confounding by study authors
						Regimen	N	Regimen	N				
Aksoylar *et al.* 1997[29]	RCT	Single teaching hospital ED, Turkey<br><br>Study period not reported	6 m to 5 y	T≥39°C (rectal), any etiology	Allergic to medication; renal, GI, hematologic, cardiopulmonary, malignant and CNS diseases; dehydration; antipyretic <6 h before study	8 mg/kg, PO, single dose	56	15 mg/kg, PO, single dose	56	Temperature at 3 h	3 h	Yes	No
Autret *et al.* 1994[30]	RCT	Pediatric wards of 14 hospitals, France<br><br>Study period not reported	6 m to 5 y	T≥38°C (rectal) due to infectious origin; need amoxycillin or co-amoxyclav in addition to antipyretic	Antipyretic ≤6 h before study; hypersensitivity to NSAIDs, aspirin, acetaminophen or penicillin; conditions that might interfere with drug absorption or distribution; severe hyperthermia with neurological and/or hemodynamic disorders; children on anti-epileptics	7.5 mg/kg, PO, first dose, then second dose given 6 h later regardless of temperature, then q6h if T>37.8°C	77	10 mg/kg, PO, first dose, then second dose given 6 h later regardless of temperature, then q6h if T>37.8°C	77	Temperature at 4 h, 12 h; Adverse events	72 h for temperature; 7 d for adverse events	Yes	No

© 2020 Tan E et al. *JAMA Network Open.*

Author and date	Study design	Setting, Country	Study population	Inclusion criteria	Exclusion criteria	Ibuprofen		Acetaminophen		Outcomes assessed	Follow-up duration	Included in meta-analysis	Adjustment for potential confounding by study authors
						Regimen	N	Regimen	N				
Autret *et al.* 1997[33]	RCT	60 pediatric private practices, France<br><br>Study period not reported	6 m to 24 m	T≥39°C (rectal)	Antipyretic ≤4 h before study; hypersensitivity to NSAIDs, aspirin, acetaminophen; conditions that might interfere with drug absorption or distribution; severe hyperthermia with neurological and/or hemodynamic disorders	7.5 mg/kg, PO, first dose, then q6h PRN, unknown duration	116	10 mg/kg, PO, first dose, then q6h PRN, unknown duration	116	Temperature at 1 h, 4 h; Pain rating (CHEOPS and facial expression) at 4 h; Adverse events	14 d	Yes	No
Erlewyn-Lajeunesse *et al.* 2006[34]	RCT	Single inner city pediatric ED, UK<br><br>Oct 2004 to Jan 2005	6 m to 10 y*	T≥38°C (tympanic)	Antipyretic ≤6 h before study; severe or life-threatening infection; suspected chickenpox; cellulitis or other spreading skin infection; shock; immunosuppressed; allergy to study drugs; on warfarin, heparin or anti-hypertensive;	5 mg/kg, PO, single dose	26	15 mg/kg, PO, single dose	26	Temperature at 1 h	Up to 2 h (if child not yet discharged)	Yes	Baseline temperature

© 2020 Tan E et al. *JAMA Network Open.*

Author and date	Study design	Setting, Country	Study population	Inclusion criteria	Exclusion criteria	Ibuprofen		Acetaminophen		Outcomes assessed	Follow-up duration	Included in meta-analysis	Adjustment for potential confounding by study authors
						Regimen	N	Regimen	N				
					active GI bleeding; known coagulopathy; acute jaundice; dehydration; asthma with regular preventer; chronic renal, liver or cardiac failure								
Hay *et al.* 2008[35]	RCT	35 primary care sites, UK<br><br>Jan 2005 to May 2007	6 m to 6 y	T≥37.8°C and up to 41°C (axillary); illness could be managed at home	Required hospital admission; dehydrated clinically; recent participation in another trial; previous participation in the trial; known intolerance, allergy or contraindication to trial drug; chronic neurological, cardiac, pulmonary (except asthma), liver, or renal disease; parents unable	10 mg/kg, PO, q6-8h, regularly from 4-24 h, then PRN from 24-48 h	52	15 mg/kg, PO, q6-8h, regularly from 4-24 h, then PRN from 24-48 h	52	Discomfort scale at 24 h, 48 h, 5 d; Temperature 2 h, 4 h, 48 h, 5 d; Adverse effects at 4 h, 24 h, 48 h, 5 d	5 d	Yes	"Factors showing possible imbalance at baseline"

© 2020 Tan E et al. *JAMA Network Open.*

Author and date	Study design	Setting, Country	Study population	Inclusion criteria	Exclusion criteria	Ibuprofen		Acetaminophen		Outcomes assessed	Follow-up duration	Included in meta-analysis	Adjustment for potential confounding by study authors
						Regimen	N	Regimen	N				
					to read or write English								
Kokki *et al.* 2010[36]	RCT	49 primary care outpatient clinics in France and UK<br><br>Study period not reported	6 m to 6 y*	Children presenting with a febrile condition requiring antipyretic; T≥38.8°C (oral) or T≥39°C (rectal); capable of taking study medication	Possible requirement for antibacterial treatment during first 6 h following baseline assessment; known contraindication to study medications; received antipyretic within 4 h or antibacterial treatment within 24 h; condition that could affect absorption of study medication such as gastroenteritis; medical conditions contraindicating use of study medication	5 mg/kg, PO, q6h for 24 h, then PRN up to 96 h	78	15 mg/kg, PO, q6h for 24 h, then PRN up to 96 h	79	Temperature at 3 h; Adverse events	96 h	Yes	No
Lesko *et al.* 1997[37]	RCT	1735 outpatient pediatric and	6 m to 12 y	Attending physician deemed	Previous participation in study; >10%	Low dose: 5 mg/kg, PO, q4-6h	Low dose: 103	12 mg/kg, PO, q4-6h PRN,	97	Renal impairment	4 w	Yes	Age, weight, sex, discharge

© 2020 Tan E et al. *JAMA Network Open.*

Author and date	Study design	Setting, Country	Study population	Inclusion criteria	Exclusion criteria	Ibuprofen		Acetaminophen		Outcomes assessed	Follow-up duration	Included in meta-analysis	Adjustment for potential confounding by study authors
						Regimen	N	Regimen	N				
		family medicine practices, USA<br><br>Feb 1991 to Jun 1993		illness warranted treatment with an antipyretic; febrile (duration and height of fever were not criteria for participation); weight 7-50 kg; able to take medication by mouth	dehydrated; unable to take medication by mouth; known sensitivity to acetaminophen, ibuprofen, aspirin, NSAID; all or part of the syndrome of nasal polys, angioedema, bronchospastic reactivity to aspirin or NSAID; chronic renal disease; blood coagulation defect; anemia secondary to blood loss; hepatic, metabolic, endocrine, neoplastic, or peptic ulcer disease	PRN, unknown duration<br><br>High dose: 10 mg/kg, PO, q4-6h PRN, unknown duration	High dose: 88	unknown duration					diagnosis (comorbidity)
Lesko *et al.* 1999[38]	RCT	1735 outpatient pediatric and family medicine	1 m to 23 m	Attending physician deemed illness warranted treatment	Previous participation in study; >10% dehydrated; unable to take medication by	Low dose: 5 mg/kg, PO, q4-6h PRN, unknown duration	Low dose: 9,159	12 mg/kg, PO, q4-6h PRN, unknown duration	9,127	Hospitalization for any reason and for specific	4 w	Yes	Age, weight, sex, dehydration

© 2020 Tan E et al. *JAMA Network Open.*

Author and date	Study design	Setting, Country	Study population	Inclusion criteria	Exclusion criteria	Ibuprofen		Acetaminophen		Outcomes assessed	Follow-up duration	Included in meta-analysis	Adjustment for potential confounding by study authors
						Regimen	N	Regimen	N				
		practices, USA<br><br>Feb 1991 to Jun 1993		with an antipyretic; febrile (duration and height of fever were not criteria for participation); weight 7-50 kg; able to take medication by mouth; parent/guardian able to administer medication according to English instructions	mouth; known sensitivity to acetaminophen, ibuprofen, aspirin, NSAID; all or part of the syndrome of nasal polys, angioedema, bronchospastic reactivity to aspirin or NSAID; chronic renal disease; blood coagulation defect; anemia secondary to blood loss; hepatic, metabolic, endocrine, neoplastic, or peptic ulcer disease	High dose: 10 mg/kg, PO, q4-6h PRN, unknown duration	High dose: 8,779			diagnoses (acute GI bleeding, acute renal failure, asthma/bronchiolitis)			
McIntyre *et al.* 1996[39]	RCT	Pediatric wards of a single hospital, UK<br><br>Study period not reported	2 m to 12 y	T≥37.5°C (axillary)	Weight <3rd centile for age; receiving anticoagulant treatment; intolerance to ibuprofen, acetaminophen or similar	5 mg/kg, PO, q6h PRN, up to 3 d	76	12.5 mg/kg, PO, q6h PRN, up to 3 d	74	Temperature at 4 h, 3 d; Irritability score at 3 d; Adverse events	3 d	Yes	Treatment, age

© 2020 Tan E et al. *JAMA Network Open.*

Author and date	Study design	Setting, Country	Study population	Inclusion criteria	Exclusion criteria	Ibuprofen		Acetaminophen		Outcomes assessed	Follow-up duration	Included in meta-analysis	Adjustment for potential confounding by study authors
						Regimen	N	Regimen	N				
					compounds; previous or current symptoms of peptic ulceration or GI bleeding; severe liver, heart, kidney or systemic disease including malignancy; medication that could interfere in the 6 h prior to study entry								
Sarrell *et al.* 2006[40]	RCT	3 (2 urban, 1 rural) primary pediatric community ambulatory centers, Israel<br><br>Sep 2003 to Mar 2004	6 m to 36 m	T≥38.4°C (rectal)	Not attending daycare; temperature-altering drugs or antibiotics within 10 d of presentation; known abnormal liver or renal laboratory values; history of renal or hepatic impairment, GI bleeding, known allergy to any antipyretic, congenital or	5 mg/kg, PO, q8h, 3 d	155	12.5 mg/kg, PO, q6h, 3 d	154	Pain rating NCCPC at 1 d, 2 d; Temperature at 1 d, 2 d; Adverse events, hepatic impairment, renal impairment, GI bleeding at 3 d, 5	10 d for fever; 12 w for adverse events	Yes	Type of loading medication had no statistical effect; patients grouped according to maintenance medication for analysis

© 2020 Tan E et al. *JAMA Network Open.*

Author and date	Study design	Setting, Country	Study population	Inclusion criteria	Exclusion criteria	Ibuprofen		Acetaminophen		Outcomes assessed	Follow-up duration	Included in meta-analysis	Adjustment for potential confounding by study authors
						Regimen	N	Regimen	N				
					acquired immunodeficiency, Reye syndrome, asthma, bronchiolitis, or malignancy; caregiver unable to apply NCCPC to measure stress					d, and 12 w			
Sheehan *et al.* 2016[31]	RCT	18 sites (type unclear; possibly outpatients), USA<br><br>Mar 2013 to Apr 2015	12 m to 59 m*	Met criteria for receiving long-term step 2 (low dose inhaled steroids, montelukast, or cromolyn) asthma-controller therapy (from National Asthma Education and Prevention Program)	History of adverse reaction to any trial medications; evidence of possible poor adherence to trial medication regimes or study procedures	9.4 mg/kg, PO, q6h PRN for 48 w	26	15 mg/kg, PO, q6h PRN for 48 w	19	Wheeze or asthma exacerbation	48 w	Yes	Length of time participants remained in the study
Van Esch *et al.* 1995[32]	RCT	Outpatient department of single teaching hospital, Netherlands	10 m to 4 y	T≥38.5°C (rectal); no contraindication to ibuprofen or	Any antipyretic or antibiotic medication within 12 h before study entry	5 mg/kg, PO, q6h PRN for 1-3 d	34	10 mg/kg, PO, q6h PRN for 1-3 d	36	Temperature at 2 h, 4 h; Adverse events	2-3 d	Yes	Initial temperature, age, weight, cause of fever

© 2020 Tan E et al. *JAMA Network Open.*

Author and date	Study design	Setting, Country	Study population	Inclusion criteria	Exclusion criteria	Ibuprofen		Acetaminophen		Outcomes assessed	Follow-up duration	Included in meta-analysis	Adjustment for potential confounding by study authors
						Regimen	N	Regimen	N				
		Jun 1991 to Oct 1993		acetaminophen									
Ashraf *et al.* 1999[41]	PC	Naturalistic outpatient general pediatric setting, USA<br><br>Mar 1993 to Jul 1995	1 m to 18 y	All-comers 1 m to 18 y<br><br>Children's Analgesic Medicine Project (CAMP)	Not stated	Usual pediatric analgesia protocols, PO	7,381	Usual pediatric analgesia protocols, PO	6,900	Serious adverse events, renal failure, GI bleeding/ perforation, necrotising fasciitis	1-2 w	Yes	Health status (sick/well) at enrolment, first use of study medication
Asl *et al.* 2015[42]	CS	Community setting, Iran<br><br>Study period not reported	6 m to 4.5 y*	Diagnosed with febrile seizure; hospitalized in pediatric ward of Shohada-e-Tajrish Hospital	Unwilling to participate; inability of parents to correctly measure body temp; <6 m or >60 m of age; history of allergy to study drugs, epilepsy, proven brain anomaly, brain surgery, or recent head trauma; anti-seizure drug prior to study; other cause for seizure during hospitalization; using 2	10 mg/kg, PO, q6h, unknown duration ? 24 h	20	15 mg/kg, PO, q6h, unknown duration ? 24 h	20	Temperature at 1 h, 4 h; Adverse events	?24 h	Yes	No

© 2020 Tan E et al. *JAMA Network Open.*

Author and date	Study design	Setting, Country	Study population	Inclusion criteria	Exclusion criteria	Ibuprofen		Acetaminophen		Outcomes assessed	Follow-up duration	Included in meta-analysis	Adjustment for potential confounding by study authors
						Regimen	N	Regimen	N				
					antipyretic drugs during febrile disease; developing adverse drug effects								
Celebi *et al.* 2009[43]	Controlled (Non-RCT)	Pediatric emergency units, Turkey<br><br>Study period not reported	6 m to 14 y*	T≥38°C (axillary) or ≥39°C (rectal) for ≥24 h due to acute infection	Chronic disease; antibiotic therapy within 1 w of presentation, antipyretic therapy within 2 d, rejection to observation for at least 4-6 h in ED, diagnosis of bacterial infection such as sepsis or meningitis that required antibiotics	10 mg/kg, PO, single dose	33	15 mg/kg, PO, single dose	50	Temperature at 1 h; Adverse events	4-6 h for temperature; 48 h for adverse events	Yes	No
Matok *et al.* 2017[44]	RCS	Single pediatric ED, Israel<br><br>Sep 2009 to Mar 2013	6 m to 6 y	T>38°C on admission or during day prior to admission; can provide data on antipyretic treatment during	Children with cystic fibrosis or chronic lung disease; ventilated on admission; parents did not speak Hebrew or Arabic; parents or	Not stated	190	Not stated	268	Wheeze	N/A	Yes	Age, atopic dermatitis, allergies, smoking, antibiotic use (data used was unadjusted)

© 2020 Tan E et al. *JAMA Network Open.*

Author and date	Study design	Setting, Country	Study population	Inclusion criteria	Exclusion criteria	Ibuprofen		Acetaminophen		Outcomes assessed	Follow-up duration	Included in meta-analysis	Adjustment for potential confounding by study authors
						Regimen	N	Regimen	N				
				current febrile illness	guardians did not consent to the study								
Sordillo *et al.* 2015[45]	PC	8 offices of Harvard Vanguard Associates in Eastern Massachusetts, USA<br><br>1999 to 2002	Birth (≥34 w) to 1 y	Live infant; ≥34 w gestation	Multiple gestation; inability to answer questions in English; plans to move out of study area before delivery of infant; gestational age <22 w at the time of presentation for prenatal care	Not stated	837	Not stated	1,165	Current asthma	10 y	No	Analgesic exposures (infancy and prenatal exposures), RTIs, ear infections, sex, multivitamin intake, maternal age, race/ethnicity, pre-pregnancy BMI, household income, no children <12y in the home, breastfeeding duration, passive smoking exposure, pregnancy smoking, childcare attendance, maternal and paternal

© 2020 Tan E et al. *JAMA Network Open.*

Author and date	Study design	Setting, Country	Study population	Inclusion criteria	Exclusion criteria	Ibuprofen		Acetaminophen		Outcomes assessed	Follow-up duration	Included in meta-analysis	Adjustment for potential confounding by study authors
						Regimen	N	Regimen	N				
													history of asthma
Souyri *et al.* 2008[12]	CC	Multiple sites in France (French Pharmaco-vigilance System), France<br><br>Jan 2000 to Dec 2004	All ages*	Cases: potential case of necrotizing soft tissue infection using WHO-ART codes describing necrotizing fasciitis - then reviewed by research team as probable or definite. Controls: 6 controls for each case, matched by gender, age and year of reporting, and excluding anyone with adverse drug reaction involving the skin	Not stated	Not stated	6	Not stated	3	Necrotising soft tissue infection	N/A	Yes	Age, sex, year of reporting to database, NSAIDs, aspirin, acetaminophen, immuno-suppresssion

© 2020 Tan E et al. *JAMA Network Open.*

Author and date	Study design	Setting, Country	Study population	Inclusion criteria	Exclusion criteria	Ibuprofen		Acetaminophen		Outcomes assessed	Follow-up duration	Included in meta-analysis	Adjustment for potential confounding by study authors
						Regimen	N	Regimen	N				
Walsh *et al.* 2018A[47]	RC	Multiple sites in California (California Medicaid Database)<br><br>2004 to 2010	Birth to 12 m	Infants for whom the Department of Health Care Services paid for at least one prescription for either acetaminophen or ibuprofen before six months of age between 2004 and 2010	Not stated	Not stated	1,724	Not stated	136,575	Acute renal impairment or failure, GI bleeding/ obstruction, necrotizing fasciitis	6 m	Yes	Age, prior history of outcomes of interest
Walsh *et al.* 2018B[46]	RC	Multiple sites in California (California Medicaid Database)<br><br>2003 to 2010	Birth to 12 m	Diagnosed with first medically attended upper respiratory infection; filled a prescription for acetaminophen or ibuprofen or both; identified using ICD-9	Diagnosis of prior or concurrent bronchiolitis or pneumonia	Not stated	8,807	Not stated	48,709	Wheeze (bronchiolitis or asthma)	1 y	Yes	Age, prior exposure, geography, previous diagnosis

© 2020 Tan E et al. *JAMA Network Open.*

Author and date	Study design	Setting, Country	Study population	Inclusion criteria	Exclusion criteria	Ibuprofen		Acetaminophen		Outcomes assessed	Follow-up duration	Included in meta-analysis	Adjustment for potential confounding by study authors
						Regimen	N	Regimen	N				
				codes 460, 465.0, 465.8, 465.9									

*data for <2 y provided by study authors
Age/time points: h = hours; d = days; w = weeks; m = months; y = years
Study design: CC = case-control; CS = cross-sectional; PC = prospective cohort; RC = retrospective cohort; RCS = retrospective cross-sectional; RCT = randomized controlled trial
BMI = body mass index
CHEOPS = Children’s Hospital of Eastern Ontario Pain Scale
CNS = central nervous system
ED = emergency department
GI = gastrointestinal
ICD = International Classification of Diseases
NCCPC = Non-Communicating Children's Pain Checklist
NSAID = non-steroidal anti-inflammatory drug
PO = *per os* (oral administration)
PRN = *pro re nata* (as needed administration)
RTI = respiratory tract infection
WHO-ART = World Health Organization Adverse Reactions Terminology

© 2020 Tan E et al. *JAMA Network Open.*

eTable 2. Risk of Bias Assessment of Included Studies

Source	Risk of bias assessment								
	Randomization process	Confounding	Selection of participants	Classification of interventions	Deviation from intended intervention	Missing data	Measurement of outcome	Reporting of results	Other risk of bias	Overall risk of bias
**Randomized**[a]										
Aksoylar et al,[29] 1997	Some concerns	NA	NA	NA	Low	High	Low	Low	NA	High risk
Autret et al,[30] 1994	Some concerns	NA	NA	NA	Low	Low	Low	Low	NA	Medium risk
Autret et al,[33] 1997	Some concerns	NA	NA	NA	Some concerns	Low	Some concerns	Low	NA	Medium risk
Erlewyn-Lajeunesse et al,[34] 2006	Low	NA	NA	NA	Some concerns	Low	Low	Low	NA	Medium risk
Hay et al,[35] 2008	Low	NA	NA	NA	Low	Low	Low	Low	NA	Low risk
Kokki et al,[36] 2010	Some concerns	NA	NA	NA	High	Low	High	Low	NA	High risk
Lesko et al,[37] 1997	Low	NA	NA	NA	Low	Low	Low	Low	NA	Low risk
Lesko et al,[38] 1999	Low	NA	NA	NA	Low	Low	Low	Some concerns	NA	Medium risk
McIntyre et al,[39] 1996	Some concerns	NA	NA	NA	Low	Low	Low	Low	NA	Medium risk
Sarrell et al,[40] 2006	Low	NA	NA	NA	Low	Low	Low	Low	NA	Low risk
Sheehan et al,[31] 2016	Low	NA	NA	NA	Low	Low	Low	Low	NA	Low risk
Van Esch et al,[32] 1995	Low	NA	NA	NA	Low	Low	Low	Low	NA	Low risk
**Nonrandomized**[b]										
Ashraf et al,[41] 1999	NA	Serious	Low	No information	No information	No information	Moderate	Moderate	NA	No information
Asl et al,[42] 2015	NA	No information	Low	Low	No information	Low	Low	Low	No analysis plan	Moderate

© 2020 Tan E et al. *JAMA Network Open.*

Celebi et al,[43] 2009	NA	No information	Low	Low	Low	Moderate	Low	Low	Critical mathematical inconsistencies	Serious
Matok et al,[44] 2017	NA	Moderate	Serious	Moderate	Low	Low	No information	Low	NA	Serious
Sordillo et al,[45] 2015	NA	Moderate	Low	Low	Moderate	No information	Moderate	Low	NA	Moderate
Souyri et al,[12] 2008	NA	Serious	Moderate	Moderate	Low	Low	Low	Low	NA	Serious
Walsh et al,[47] 2018A	NA	Serious	Serious	Serious	No information	Serious	Moderate	Low	NA	Serious
Walsh et al,[46] 2018B	NA	Moderate	Low	Low	Low	Low	Low	Low	NA	Moderate

Abbreviation: NA, not applicable.

[a]Risk of bias for randomized studies assessed with Cochrane Risk of Bias 2 tool.[26]

[b]Risk of bias for nonrandomized studies assessed with ROBINS-I tool.[27]

© 2020 Tan E et al. *JAMA Network Open.*

eFigure 1. Antipyretic Profile (Continuous Variable) of Ibuprofen vs Acetaminophen From Nonrandomized Studies

	Acetaminophen			Ibuprofen				Std. Mean Difference
**Study or Subgroup**	**Mean**	**SD**	**Total**	**Mean**	**SD**	**Total**	**Weight**	**IV, Random, 95% CI**
**1.1.1 Temperature or change in temperature at <4h**								
Asl 2015 (1)	−0.47	0.33	20	−0.41	0.38	20	33.4%	−0.17 [−0.79, 0.46]
Celebi 2009 (2)	−0.74	0.56	50	−0.75	0.62	33	66.6%	0.02 [−0.42, 0.46]
**Subtotal (95% CI)**			**70**			**53**	**100.0%**	**−0.04 [−0.40, 0.31]**
Heterogeneity: Tau^2 = 0.00; Chi^2 = 0.22, df = 1 (P = 0.64); I^2 = 0%								
Test for overall effect: Z = 0.24 (P = 0.81)								
**1.1.2 Temperature or change in temperature at 4–24h**								
Asl 2015 (3)	−0.76	0.36	20	−0.83	0.42	20	100.0%	0.18 [−0.45, 0.80]
**Subtotal (95% CI)**			**20**			**20**	**100.0%**	**0.18 [−0.45, 0.80]**
Heterogeneity: Not applicable								
Test for overall effect: Z = 0.55 (P = 0.58)								
**1.1.3 Temperature or change in temperature at 1–3d**								
**Subtotal (95% CI)**			**0**			**0**		**Not estimable**
Heterogeneity: Not applicable								
Test for overall effect: Not applicable								
**1.1.4 Temperature or change in temperature at >3d**								
**Subtotal (95% CI)**			**0**			**0**		**Not estimable**
Heterogeneity: Not applicable								
Test for overall effect: Not applicable								

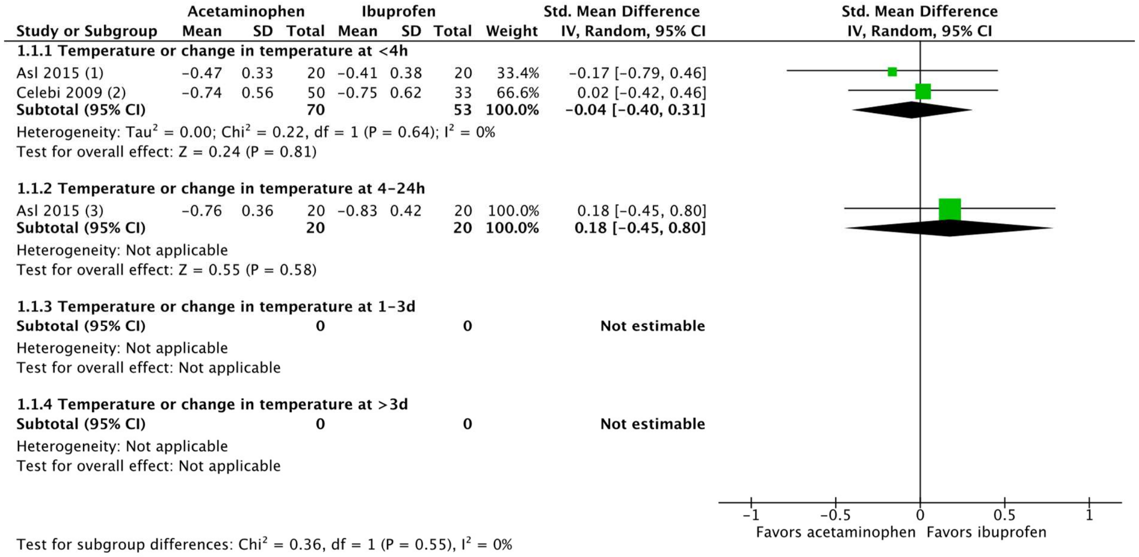

Test for subgroup differences: Chi^2 = 0.36, df = 1 (P = 0.55), I^2 = 0%

Footnotes
(1) Mean fever decrease 1h
(2) Mean antipyretic effect 1h
(3) Mean fever decrease 4h

© 2020 Tan E et al. *JAMA Network Open.*

eFigure 2. Subgroup Analyses of Antipyretic Profile Within 4 Hours Comparing Lower and Higher Dosages of Ibuprofen vs Acetaminophen

(a) Randomized studies

Study or Subgroup	Acetaminophen Mean	SD	Total	Ibuprofen Mean	SD	Total	Weight	Std. Mean Difference IV, Fixed, 95% CI
**1.6.1 High dose paracetamol (>10mg/kg) vs high dose ibuprofen (>5mg/kg)**								
Aksoylar 1997 (1)	38.4	0.71	51	37.9	0.71	50	100.0%	0.70 [0.30, 1.10]
**Subtotal (95% CI)**			**51**			**50**	**100.0%**	**0.70 [0.30, 1.10]**
Heterogeneity: Not applicable								
Test for overall effect: Z = 3.41 (P = 0.0007)								
**1.6.2 Low dose paracetamol (≤10mg/kg) vs high dose ibuprofen (>5mg/kg)**								
Autret 1997 (2)	−0.9	0.56	114	−0.97	0.58	114	100.0%	0.12 [−0.14, 0.38]
**Subtotal (95% CI)**			**114**			**114**	**100.0%**	**0.12 [−0.14, 0.38]**
Heterogeneity: Not applicable								
Test for overall effect: Z = 0.92 (P = 0.36)								
**1.6.3 High dose paracetamol (>10mg/kg) vs low dose ibuprofen (≤5mg/kg)**								
Erlewyn-Lajeunesse 2006 (3)	37.95	0.48	25	37.76	0.62	22	100.0%	0.34 [−0.24, 0.92]
**Subtotal (95% CI)**			**25**			**22**	**100.0%**	**0.34 [−0.24, 0.92]**
Heterogeneity: Not applicable								
Test for overall effect: Z = 1.15 (P = 0.25)								
**1.6.4 Low dose paracetamol (≤10mg/kg) vs low dose ibuprofen (≤5mg/kg)**								
Van Esch 1995 (4)	37.96	0.92	29	37.6	0.6	30	100.0%	0.46 [−0.06, 0.98]
**Subtotal (95% CI)**			**29**			**30**	**100.0%**	**0.46 [−0.06, 0.98]**
Heterogeneity: Not applicable								
Test for overall effect: Z = 1.74 (P = 0.08)								

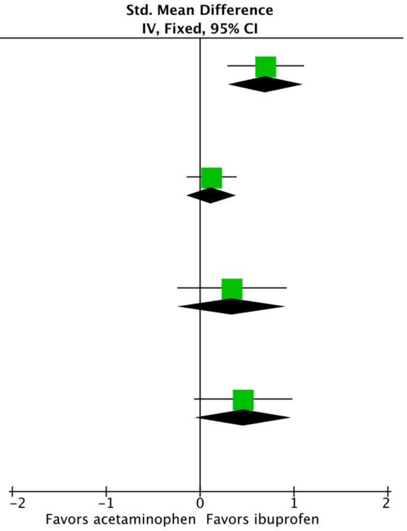

Test for subgroup differences: $Chi^2 = 5.91$, df = 3 (P = 0.12), $I^2 = 49.2\%$

Footnotes
(1) Temperature 3h
(2) Mean reduction in temperature 1h
(3) Mean temperature 1h
(4) Mean temperature 2h

(b) Nonrandomized studies

Study or Subgroup	Acetaminophen Mean	SD	Total	Ibuprofen Mean	SD	Total	Weight	Std. Mean Difference IV, Fixed, 95% CI
**1.7.1 High dose paracetamol (>10mg/kg) vs high dose ibuprofen (>5mg/kg)**								
Asl 2015 (1)	−0.47	0.33	20	−0.41	0.38	20	33.4%	−0.17 [−0.79, 0.46]
Celebi 2009 (2)	−0.74	0.56	50	−0.75	0.62	33	66.6%	0.02 [−0.42, 0.46]
**Subtotal (95% CI)**			**70**			**53**	**100.0%**	**−0.04 [−0.40, 0.31]**
Heterogeneity: $Chi^2 = 0.22$, df = 1 (P = 0.64); $I^2 = 0\%$								
Test for overall effect: Z = 0.24 (P = 0.81)								
**1.7.2 Low dose paracetamol (≤10mg/kg) vs high dose ibuprofen (>5mg/kg)**								
**Subtotal (95% CI)**			**0**			**0**		**Not estimable**
Heterogeneity: Not applicable								
Test for overall effect: Not applicable								
**1.7.3 High dose paracetamol (>10mg/kg) vs low dose ibuprofen (≤5mg/kg)**								
**Subtotal (95% CI)**			**0**			**0**		**Not estimable**
Heterogeneity: Not applicable								
Test for overall effect: Not applicable								
**1.7.4 Low dose paracetamol (≤10mg/kg) vs low dose ibuprofen (≤5mg/kg)**								
**Subtotal (95% CI)**			**0**			**0**		**Not estimable**
Heterogeneity: Not applicable								
Test for overall effect: Not applicable								

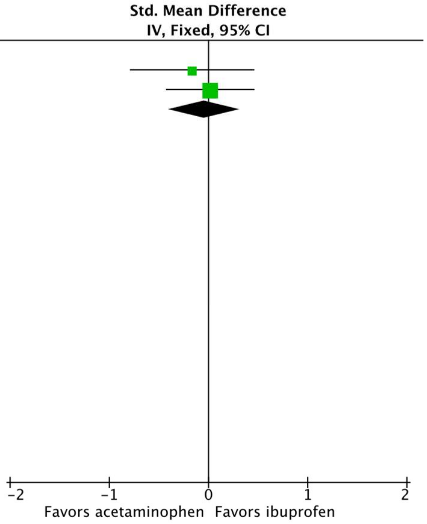

Test for subgroup differences: Not applicable

Footnotes
(1) Mean fever decrease 1h
(2) Mean antipyretic effect 1h

© 2020 Tan E et al. *JAMA Network Open.*

eFigure 3. Adverse Events With Ibuprofen vs Acetaminophen From Randomized Studies

Study or Subgroup	Ibuprofen Events	Ibuprofen Total	Acetaminophen Events	Acetaminophen Total	Weight	Odds Ratio IV, Fixed, 95% CI
**1.5.1 Serious adverse events (fatal, life-threatening, hospitalisation) ≤28d**						
Autret 1994 (1)	0	77	0	77		Not estimable
Autret 1997 (2)	0	116	0	116		Not estimable
Hay 2008 (3)	3	52	1	52	0.9%	3.12 [0.31, 31.05]
Kokki 2010 (4)	0	78	1	79	0.4%	0.33 [0.01, 8.31]
Lesko 1999 (5)	261	17938	124	9127	98.7%	1.07 [0.86, 1.33]
McIntyre 1996 (6)	0	76	0	74		Not estimable
Van Esch 1995 (7)	0	34	0	36		Not estimable
**Subtotal (95% CI)**		**18371**		**9561**	**100.0%**	**1.08 [0.87, 1.33]**
Total events	264		126			
Heterogeneity: Chi² = 1.34, df = 2 (P = 0.51); I² = 0%						
Test for overall effect: Z = 0.67 (P = 0.50)						
**1.5.2 Renal impairment ≤28d**						
Kokki 2010 (8)	0	78	0	79		Not estimable
Lesko 1997 (9)	15	155	6	67	64.3%	1.09 [0.40, 2.94]
Lesko 1999 (10)	0	17938	0	9127		Not estimable
Sarrell 2006 (11)	4	155	5	154	35.7%	0.79 [0.21, 3.00]
**Subtotal (95% CI)**		**18326**		**9427**	**100.0%**	**0.97 [0.44, 2.15]**
Total events	19		11			
Heterogeneity: Chi² = 0.14, df = 1 (P = 0.70); I² = 0%						
Test for overall effect: Z = 0.07 (P = 0.94)						
**1.5.3 GI bleeding ≤28d**						
Kokki 2010 (12)	0	78	0	79		Not estimable
Lesko 1999 (13)	3	17938	0	9127	100.0%	3.56 [0.18, 68.97]
Sarrell 2006 (14)	0	155	0	154		Not estimable
**Subtotal (95% CI)**		**18171**		**9360**	**100.0%**	**3.56 [0.18, 68.97]**
Total events	3		0			
Heterogeneity: Not applicable						
Test for overall effect: Z = 0.84 (P = 0.40)						
**1.5.4 Hepatotoxicity ≤28d**						
Kokki 2010 (15)	0	78	0	79		Not estimable
Sarrell 2006 (16)	2	155	4	154	100.0%	0.49 [0.09, 2.72]
**Subtotal (95% CI)**		**233**		**233**	**100.0%**	**0.49 [0.09, 2.72]**
Total events	2		4			
Heterogeneity: Not applicable						
Test for overall effect: Z = 0.82 (P = 0.41)						
**1.5.5 Severe soft tissue infection ≤28d**						
Kokki 2010 (17)	0	78	0	79		Not estimable
**Subtotal (95% CI)**		**78**		**79**		**Not estimable**
Total events	0		0			
Heterogeneity: Not applicable						
Test for overall effect: Not applicable						
**1.5.6 Empyema ≤28d**						
Kokki 2010 (18)	0	78	0	79		Not estimable
**Subtotal (95% CI)**		**78**		**79**		**Not estimable**
Total events	0		0			
Heterogeneity: Not applicable						
Test for overall effect: Not applicable						
**1.5.7 Asthma or wheeze ≤28d**						
Kokki 2010 (19)	0	78	0	79		Not estimable
Lesko 1999 (20)	41	17938	24	9127	97.3%	0.87 [0.52, 1.44]
McIntyre 1996 (21)	0	76	2	74	2.7%	0.19 [0.01, 4.02]
**Subtotal (95% CI)**		**18092**		**9280**	**100.0%**	**0.83 [0.51, 1.37]**
Total events	41		26			
Heterogeneity: Chi² = 0.93, df = 1 (P = 0.33); I² = 0%						
Test for overall effect: Z = 0.71 (P = 0.48)						
**1.5.8 Serious adverse events (fatal, life-threatening, hospitalisation) >28d**						
Sarrell 2006 (22)	0	155	0	154		Not estimable
Sheehan 2016	0	26	0	19		Not estimable
**Subtotal (95% CI)**		**181**		**173**		**Not estimable**
Total events	0		0			
Heterogeneity: Not applicable						
Test for overall effect: Not applicable						
**1.5.9 Renal impairment >28d**						
Sarrell 2006 (23)	0	155	0	154		Not estimable
Sheehan 2016	0	26	0	19		Not estimable
**Subtotal (95% CI)**		**181**		**173**		**Not estimable**
Total events	0		0			
Heterogeneity: Not applicable						
Test for overall effect: Not applicable						
**1.5.10 GI bleeding >28d**						
Sarrell 2006 (24)	0	155	0	154		Not estimable
Sheehan 2016	0	26	0	19		Not estimable
**Subtotal (95% CI)**		**181**		**173**		**Not estimable**
Total events	0		0			
Heterogeneity: Not applicable						
Test for overall effect: Not applicable						
**1.5.11 Hepatotoxicity >28d**						
Sarrell 2006 (25)	0	155	0	154		Not estimable
Sheehan 2016	0	26	0	19		Not estimable
**Subtotal (95% CI)**		**181**		**173**		**Not estimable**
Total events	0		0			
Heterogeneity: Not applicable						
Test for overall effect: Not applicable						
**1.5.12 Severe soft tissue infection >28d**						
Sheehan 2016	0	26	0	19		Not estimable
**Subtotal (95% CI)**		**26**		**19**		**Not estimable**
Total events	0		0			
Heterogeneity: Not applicable						
Test for overall effect: Not applicable						
**1.5.13 Empyema >28d**						
Sheehan 2016	0	26	0	19		Not estimable
**Subtotal (95% CI)**		**26**		**19**		**Not estimable**
Total events	0		0			
Heterogeneity: Not applicable						
Test for overall effect: Not applicable						
**1.5.14 Asthma or wheeze >28d**						
Sheehan 2016	15	26	12	19	100.0%	0.80 [0.24, 2.68]
**Subtotal (95% CI)**		**26**		**19**	**100.0%**	**0.80 [0.24, 2.68]**
Total events	15		12			
Heterogeneity: Not applicable						
Test for overall effect: Z = 0.37 (P = 0.71)						

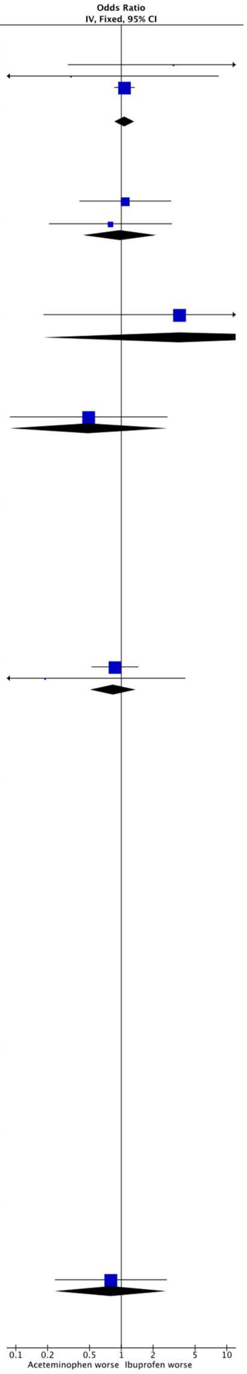

Test for subgroup differences: Chi² = 2.43, df = 5 (P = 0.79), I² = 0%

Footnotes
(1) Serious adverse events 7d
(2) Serious adverse events 14d
(3) Hospitalization 5d
(4) Hospitalization up to 96h
(5) Hospitalization for any reason up to 4w
(6) Adverse events 3d
(7) Adverse events 3d
(8) Renal impairment up to 96h
(9) Number with creatinine >62mcmol/L up to 4w
(10) Hospitalization for acute renal failure up to 4w
(11) Abnormal renal laboratory values "acute stage" up to 5d
(12) GI bleeding up to 96h
(13) Hospitalization for acute GI bleeding up to 4w
(14) Positive FOB test 3d, 5d, q2weekly up to 12w
(15) Hepatotoxicity up to 96h
(16) Abnormal liver laboratory values "acute stage" up to 5d
(17) Severe soft tissue infection up to 96h
(18) Empyema up to 96h
(19) Asthma or wheeze up to 96h
(20) Hospitalization for asthma/bronchiolitis up to 4w
(21) Wheezing 3d
(22) Drug-related serious illness up to 12w
(23) Abnormal renal function test up to 12w
(24) Positive FOB test 3d, 5d, q2weekly up to 12w
(25) Abnormal liver function test up to 12w

© 2020 Tan E et al. *JAMA Network Open.*

eFigure 4. Adverse Events With Ibuprofen vs Acetaminophen From Nonrandomized Studies

Study or Subgroup	log[Odds Ratio]	SE	Weight	Odds Ratio IV, Fixed, 95% CI
**1.5.1 Serious adverse events (fatal, life-threatening, hospitalisation) ≤28d**				
Ashraf 1999 (1)	0	0		Not estimable
Celebi 2009 (2)	0	0		Not estimable
**Subtotal (95% CI)**				**Not estimable**
Heterogeneity: Not applicable				
Test for overall effect: Not applicable				
**1.5.2 Renal impairment ≤28d**				
Ashraf 1999 (3)	0	0		Not estimable
**Subtotal (95% CI)**				**Not estimable**
Heterogeneity: Not applicable				
Test for overall effect: Not applicable				
**1.5.3 GI bleeding ≤28d**				
Ashraf 1999 (4)	0	0		Not estimable
**Subtotal (95% CI)**				**Not estimable**
Heterogeneity: Not applicable				
Test for overall effect: Not applicable				
**1.5.4 Hepatotoxicity ≤28d**				
**Subtotal (95% CI)**				**Not estimable**
Heterogeneity: Not applicable				
Test for overall effect: Not applicable				
**1.5.5 Severe soft tissue infection ≤28d**				
Ashraf 1999 (5)	0	0		Not estimable
Souyri 2008 (6)	2.5337	1.7053	100.0%	12.60 [0.45, 356.39]
**Subtotal (95% CI)**			**100.0%**	**12.60 [0.45, 356.39]**
Heterogeneity: Not applicable				
Test for overall effect: Z = 1.49 (P = 0.14)				
**1.5.6 Empyema ≤28d**				
**Subtotal (95% CI)**				**Not estimable**
Heterogeneity: Not applicable				
Test for overall effect: Not applicable				
**1.5.7 Asthma or wheeze ≤28d**				
Matok 2017 (7)	0.4055	0.7882	3.4%	1.50 [0.32, 7.03]
Walsh 2018B (8)	−0.0346	0.1478	96.6%	0.97 [0.72, 1.29]
**Subtotal (95% CI)**			**100.0%**	**0.98 [0.74, 1.30]**
Heterogeneity: Chi^2 = 0.30, df = 1 (P = 0.58); I^2 = 0%				
Test for overall effect: Z = 0.14 (P = 0.89)				
**1.5.8 Serious adverse events (fatal, life-threatening, hospitalisation) >28d**				
Asl 2015 (9)	0	0		Not estimable
**Subtotal (95% CI)**				**Not estimable**
Heterogeneity: Not applicable				
Test for overall effect: Not applicable				
**1.5.9 Renal impairment >28d**				
Walsh 2018A (10)	−0.4407	1.4202	100.0%	0.64 [0.04, 10.41]
**Subtotal (95% CI)**			**100.0%**	**0.64 [0.04, 10.41]**
Heterogeneity: Not applicable				
Test for overall effect: Z = 0.31 (P = 0.76)				
**1.5.10 GI bleeding >28d**				
Walsh 2018A (11)	0.0092	0.4116	100.0%	1.01 [0.45, 2.26]
**Subtotal (95% CI)**			**100.0%**	**1.01 [0.45, 2.26]**
Heterogeneity: Not applicable				
Test for overall effect: Z = 0.02 (P = 0.98)				
**1.5.11 Hepatotoxicity >28d**				
**Subtotal (95% CI)**				**Not estimable**
Heterogeneity: Not applicable				
Test for overall effect: Not applicable				
**1.5.12 Severe soft tissue infection >28d**				
Walsh 2018A (12)	0	0		Not estimable
**Subtotal (95% CI)**				**Not estimable**
Heterogeneity: Not applicable				
Test for overall effect: Not applicable				
**1.5.13 Empyema >28d**				
**Subtotal (95% CI)**				**Not estimable**
Heterogeneity: Not applicable				
Test for overall effect: Not applicable				
**1.5.14 Asthma or wheeze >28d**				
Walsh 2018B (13)	−0.1839	0.0773	100.0%	0.83 [0.72, 0.97]
**Subtotal (95% CI)**			**100.0%**	**0.83 [0.72, 0.97]**
Heterogeneity: Not applicable				
Test for overall effect: Z = 2.38 (P = 0.02)				

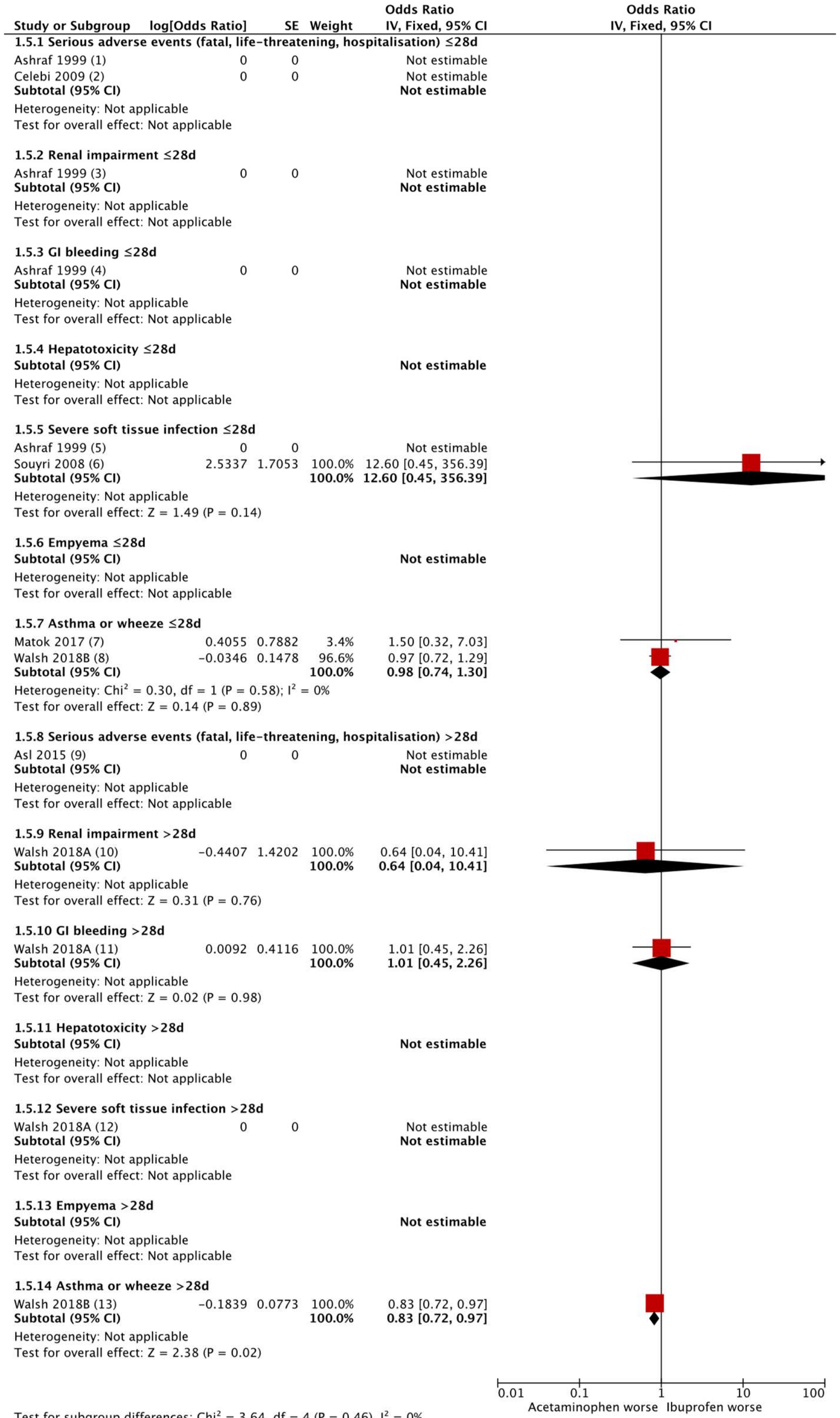

Test for subgroup differences: Chi^2 = 3.64, df = 4 (P = 0.46), I^2 = 0%

Footnotes

(1) Serious adverse events (raw data)
(2) Adverse events (raw data)
(3) Renal failure (raw data)
(4) GI bleeding or perforation (raw data)
(5) Necrotising fasciitis (raw data)
(6) Necrotising soft tissue infection (timepoint not stated)(raw data)
(7) Wheezing (raw data)
(8) Subsequent doctor visits at 14d (adjusted)
(9) Serious adverse effects (raw data)
(10) Acute renal impairment or failure (raw data)
(11) GI bleeding or obstruction (raw data)
(12) Necrotising fasciitis (raw data)
(13) Subsequent doctor visits at 365d for wheezing (adjusted)

© 2020 Tan E et al. *JAMA Network Open.*
